# Stock collapse and its effect on species interactions: Cod and herring in the Norwegian‐Barents Seas system as an example

**DOI:** 10.1002/ece3.8336

**Published:** 2021-12-01

**Authors:** Joël M. Durant, Leana Aarvold, Øystein Langangen

**Affiliations:** ^1^ Centre for Ecological and Evolutionary Synthesis (CEES) Department of Biosciences University of Oslo Oslo Norway; ^2^ Section for Aquatic biology and toxicology (AQUA) Department of Biosciences University of Oslo Oslo Norway

**Keywords:** climate effects, cod, collapse, demographic effects, herring, population dynamics

## Abstract

Both the Norwegian Spring Spawning herring (*Clupea harengus*) and the Northeast Arctic (NEA) cod (*Gadus morhua*) are examples of strong stock reduction and decline of the associated fisheries due to overfishing followed by a recovery. Cod and herring are both part of the Barents Sea ecosystem, which has experienced major warming events in the early (1920–1940) and late 20th century. While the collapse or near collapse of these stocks seems to be linked to an instability created by overfishing and climate, the difference of population dynamics before and after is not fully understood. In particular, it is unclear how the changes in population dynamics before and after the collapses are associated with biotic interactions. The combination of the availability of unique long‐term time series for herring and cod makes it a well‐suited study system to investigate the effects of collapse. We examine how species interactions may differently affect the herring and cod population dynamic before and after a collapse. Particularly we explore, using a GAM modeling approach, how herring could affect cod and *vice versa*. We found that the effect of cod biomass on herring that was generally positive (i.e., covariation) but the effect became negative after the collapse (i.e., predation or competition). Likewise a change occurred for the cod, the juvenile herring biomass that had no effect before the collapse had a negative effect after. Our results indicate that the population collapses may alter the inter‐specific interactions and response to abiotic environmental changes. While the stocks are at similar abundance levels before and after the collapses, the system is potentially different in its functioning and may require different management action.

## Graphical abstract

Stock collapses not only change abundance and the relationship with environment conditions but also the long‐term interaction with other species. We illustrated this with two iconic stocks the Norwegian Spring Spawning herring and the Northeast Arctic cod.

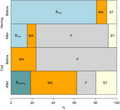

## 1 INTRODUCTION

At the stock level, the Norwegian Spring Spawning (NSS) herring (*Clupea harengus*) is a text book example of overexploitation of marine fish populations with a positive outcome. Due to overexploitation, the NSS herring stock collapsed in the 1960s from a biomass of more than 14 million tonnes in 1956 to less than 0.1 million tonnes in 1972 (Toresen & Østvedt, 2000), but is now counted as one of the largest herring stocks in the world (Engelhard & Heino, 2004). At the species level, the Atlantic cod (*Gadus morhua*) is a prime example for the overexploitation of marine fish populations (Sguotti et al., 2019), with the Northern cod collapse in the early 1990s being a major example of a stock collapse without a recovery even after the introduction of a fishing moratorium (Bundy et al., 2009; Hutchings & Rangeley, 2011). On the other hand, the Northeast Arctic (NEA) cod, despite having experienced a major decline in abundance (hereafter referred to as a collapse) in the early 1980s (Hylen, 2002) is currently at an historically high biomass and supports a healthy fishery (about 849,000 tonnes in 2016; ICES, 2017).

**FIGURE 1:**
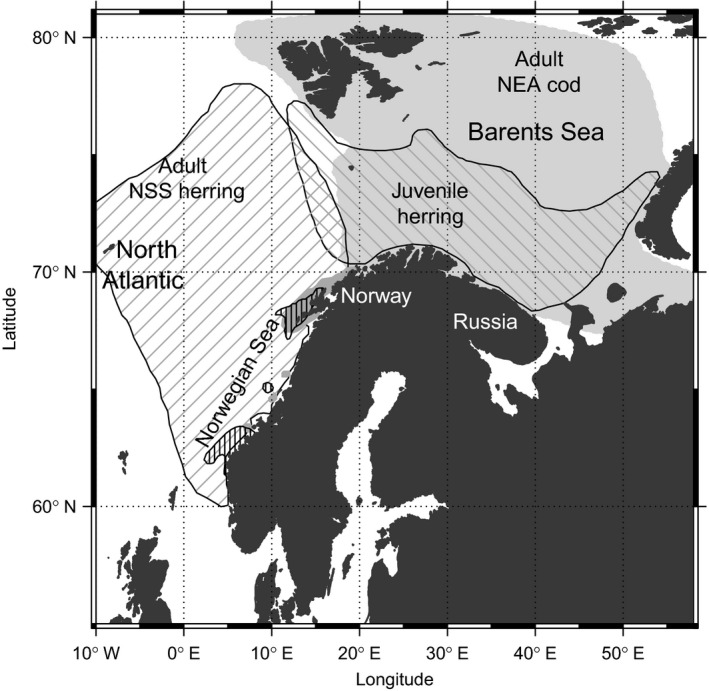
General distribution of the Northeast Arctic cod *Gadus morhua* and the Norwegian spring spawning herring *Clupea harengus* in the Norwegian Sea—Barents Sea system. The major spawning grounds for herring (vertical dashed lines) and cod (dark grey) are indicated along the coast of Norway. Map based on an Institute of Marine Research in Bergen report from 2016 (Bakketeig et al., 2016)

Abrupt and unexpected transitions between alternative system states are often the consequence of climate change, overexploitation or a combination of both (Benson & Trites, 2002; Daskalov, 2002; Hare & Mantua, 2000). Commercially exploited fish species that experienced population collapse are prominent examples with important socio‐economic ramifications (Beisner et al., 2003; Frank et al., 2011; Myers & Worm, 2003; Steneck & Wahle, 2013). Studying the population dynamic and the effect of the population structure on the population change of two co‐occurring fish in the Norwegian sea‐Barents Sea system (Figure 1), Rouyer et al. (2011) concluded that the NSS herring and NEA cod stocks were responding to environmental forcing differently before compared to after the collapses. Studying 11 exploited stocks, Durant and Hjermann (2017) showed that the population growth dependence on reproduction was linked to the stock structure and hence likely the level of fishing (Law, 2000; Ottersen et al., 2006).

While stock collapses seem to be linked to instability created by overfishing in conjunction to climate (Sguotti et al., 2019), the difference of dynamics before and after a major abundance, decline is less well understood. Although Rouyer et al. (2011) previously described the effect of collapse on both NEA cod and NSS herring stocks, they did not consider the interactions between them. Here, we hypothesis that a collapse, in addition to modifying the response to harvesting intensity and climate forcing, will change the trophic interactions. Both NEA cod and NSS herring, for the latter up to 3–4 years of age as the adults living in the North Atlantic, are part of the Barents Sea ecosystem and can interact. For instance, Minto and Worm (2012), investigating the relationships between small pelagic fish and Atlantic cod recruitment throughout the North Atlantic. They found predominantly negative relationships between herring abundance and cod recruitment, suggesting that herring has a consistently negative effect on cod recruitment (albeit not for the Norwegian‐Barents Seas system). In the North Sea, Hjermann et al. (2013) show that herring may compete with young cod for food and may in turn be preyed upon by large cod in a reversal of dominance pattern (Fauchald, 2010). In the Barents Sea, Holt et al. showed that cod are feeding on herring using stomach content data (Holt et al., 2019). Here, we hypothesize that the stock collapses may have changed how the abundance of young NSS herring affects the NEA cod dynamic and how the NEA cod abundance is affecting the NSS herring dynamic.

The Barents Sea ecosystem experienced major warming events in the early (1920–1940) and late (1975‐ongoing) 20th century (Bengtsson et al., 2004). Sea temperature fluctuations are mainly governed by atmospheric forcing and climate cycles. One such driver in the North Atlantic is the North Atlantic Oscillation index (NAO, Hurrell & Deser, 2009), which captures complex spatio‐temporal variability into a simple metric and integrates larger scale climate processes and their variability (Hallett et al., 2004). It has been documented that the NAO for winter months (wNAO, December–March) can affect different organisms in the Barents Sea such as the NEA cod (Hjermann et al., 2004) and the NSS herring (Tiedemann et al., 2021). Sea temperature affects the fish early life stages in the Barents Sea though survival and growth (Dingsør et al., 2007), distribution (Hidalgo et al., 2012), and recruitment (Ottersen et al., 2013). In the Barents Sea, the Kola transect sea temperature (ST, Bochkov, 1982; Tereschenko, 1996) is representative of the Atlantic water masses in the south‐central Barents Sea (Ingvaldsen et al., 2003) and explains the dynamics for NEA cod (Hjermann et al., 2004) and development of both cod and herring (Ottersen & Loeng, 2000). Kola transect sea temperature correlates positively with strong year‐classes of NEA cod (reviewed by Ottersen et al., 2014). Both wNAO and sea temperature have previously been used as environmental drivers to explain population growth in the Barents Sea (Durant & Hjermann, 2017; Rouyer et al., 2011).

To understand how stock collapse may affect population dynamic, we compared the population dynamic of stocks before and after a collapse. The combination of unique long‐term time series for NSS herring and NEA cod in an ecosystem that experienced large fluctuations of temperature and fishing pressure constitutes a well‐suited case study to investigate the effects of collapse. To have a common metric directly comparable, we calculated the dominant eigenvalues (de Kroon et al., 1986) of age‐structured transition matrices (Brosset et al., 2019; Durant & Hjermann, 2017; Durant et al., 2008; Rouyer et al., 2011) summarizing the vital rates of the population (survival and reproduction) and representing a proxy for the year to year population changes in one value. This allows us to examine how a collapse could alter the effect of the age structure, the fishing mortality, sea temperature, the North Atlantic oscillation, and/or interacting species on the annual change in the dominant eigenvalue.

## 2 MATERIALS AND METHODS

To understand how stock collapse may affect the population dynamic, we modeled the change of the log‐transformed dominant eigenvalue *λ*_1_ of the annual transition matrices in two interacting stocks for the periods before and after a collapse. For each period, we related the temporal variability in each stock ln(*λ*_1_) to demographic variables such as fishing mortality and mean age of the spawning stock, as well as to environmental variables (both regional and large‐scale climate indices) and potential interacting species (age 1–3 herring biomass, cod spawning stock (i.e., large cod) biomass; Table 1). The rationale for looking at the effect of environmental variables, which only indirectly affect the realized annual population growth rate, is that these variables may influence several demographic variables simultaneously, including recruitment and age of maturity (Rouyer et al., 2011).

**TABLE 1:** Data source

Stock	Period^a^	Data		Source
NEA cod	1913–1999 (1921–1973)	Fishing mortality (*F*_5–10_) Maturity at age (%) Number at age (10^3^) Biomass (10^5^ t)		Hylen (2002), Rouyer et al. (2011) Hylen (2002), Rouyer et al. (2011) Hylen (2002), Rouyer et al. (2011) Hylen (2002), Rouyer et al. (2011)
NEA cod	1946–2016 (1981–2013)	Fishing mortality (*F*_5–10_) Maturity at age (%) Number at age (10^3^) Biomass (10^3^ t)	Table 3.18 p 160 Table 3.11 p 147 Table 3.16 p 156 Table 3.18 p 160	ICES (2017) ICES (2017) ICES (2017) ICES (2017)
NSS herring	1907–1998 (1921–1964)	Fishing mortality (*F*_5–12_) Maturity at age (%) Number at age (10^6^) Biomass (10^3^ t)	Table 7 p 245 Table 5 p 242 Table 6 p 243 Table 8 p 248 (Total ‐ SSB)	Toresen and Østvedt (2000) Toresen and Østvedt (2000) Toresen and Østvedt (2000) Toresen and Østvedt (2000)
NSS herring	1950–2015 (1974–2011)	Fishing mortality (*F*_5–12_)	Table 3.4.2 p 66 + Table 7.6.2.3.2 p 463	ICES (2017) + ICES (2015)
		Maturity at age (%)	Table 7.4.5.1 p 451	ICES, 2015
		Number at age (10^9^)	Table 3.4.1 p 64 + Table 7.6.2.3.1 p 462	ICES (2017) + ICES (2015)
		Biomass (10^6^ t)	Table 7.6.2.3.3 p 464 (Total ‐ SSB)	ICES (2015)

Abbreviations: NEA, Northeast Arctic; NSS, Norwegian Spring Spawning; SSB, Spawning Stock Biomass.

^a^ Maximum year period covered by the data. The years used for the different GAM analyses are given between parentheses. Virtual population analysis (VPA) data being not reliable in the later years we used a shorter time series than available.

### 2.1 Data

The data are model‐based estimates derived from a form of Virtual Population Analysis (VPAs). These analyses are based on data from commercial catches, calibrated with fisheries' independent survey data and combined with estimates of natural mortality. Table 1 summarizes sources of the data used.

Data for NEA cod (*Gadus morhua*) were obtained from the long‐term VPA performed by Hylen (2002) over the period 1913–1999 and from the International Council for the Exploration of the Sea (ICES) VPA performed over the period 1946–2016 (ICES, 2017).

Data for NSS herring (*Clupea harengus*) were obtained from the VPA performed by Toresen and Østvedt (2000) over the period 1907–1998 and from the ICES VPA performed over the period 1950–2015 (ICES, 2007, 2015). The consistency of the different abundance sources was confirmed by Rouyer et al. (2011) by comparing the time series of abundance of each age class in the overlapping years (1946–2000 for cod and 1988–1997 for herring).

Since annual values on the proportion of maturity at age was only available from 1950 onward; we extended the ICES matrix of maturity at age for the period 1907–1949 by replicating the year 1950 (using instead average maturity values for the years before the collapse, that is, 1950–1964 see later, did not lead to a significant difference of *λ*_1_).

To define the period of collapse, we calculated the 50% quantile of ln(*λ*_1_) for each time series separately (see below for calculation of ln(*λ*_1_)). We then located the year when ln(*λ*_1_) passed the 50% quantile at the end of the time series for the data covering the period before the collapse and at the beginning of the time series for the data covering the period after the collapse. Using this method, the collapse period for the herring was 1965–1973 and for the cod 1974–1980. We analyzed the data outside the collapse periods: in 1921–1964 and 1974–2011 for the herring and 1921–1973 and 1981–2013 for the cod.

### 2.2 Dependent variable ln(*λ*_1_)

To allow for a direct comparison between the two studied stocks while integrating the maximum information, that is, spawning biomass, recruitment, abundance, and maturity, from the complex age‐structured dynamics of NEA cod and NSS herring, we summarized the vital rates (time‐ and age‐specific survival, fecundity, and recruitment success) in an annual transition matrix *A_t_* (matrix *A* at time *t*). By definition, the population size in the following year (*n_t_*_+1_) is the product of the matrix *A_t_* and the current year's population size (*n_t_*), where the “*n*” are vectors representing the number of individuals for each age class. For each year *t*, the transition matrix *A_t_* is defined as follows:

$$A_{t}=\left[\begin{array}{ccccc} R_{1,t} & R_{2,t} & R_{3,t} & \cdots & R_{amax,t}\\ S_{1‐0,t} & o & o & \cdots & o\\ o & S_{2‐1,t} & o & \cdots & o\\ \cdots & \cdots & \cdots & \cdots & \cdots \\ o & o & o & S_{amax‐(amax‐1),t} & o \end{array}\right]$$

with *R_a_*_,_ *_t_* the contribution of each age‐class *a* to the recruitment at year *t* and with *S_a_*_−(_ *_a_*_−1),_ *_t_* the survival of age *a* − 1 at year *t* to age *a* at year *t* + 1, computed as the ratio of the abundance of age *a* at year *t* and age *a* − 1 at year *t* − 1. The age index *a* varies between 1 and *a*max, the older age‐class in the population (respectively *a*max = 12 years and *a*max = 14 years for cod and herring). Note that for both stocks, the last age classes (15+ for herring and 13+ for cod) were ignored in order not to get an unrealistic survival *S_a_*_max–(_ *_a_*_max−1)_ > 1. *S* been directly calculated through abundance estimates, *S_a_*_−(_ *_a_*_−1)_ can be approximated to exp(−(*M_a_*_−(_ *_a_*_−1)_ + *F_a_*_−(_ *_a_*_−1)_)) with *M* the natural mortality and *F* the fishing mortality for age *a*.

*R_a_*_,_ *_t_* the contribution of each age‐class *a* to the recruitment at year *t* was defined as follows:

$$R_{a,t}=\frac{{\text{Rec}}_{t}{\text{MAT}}_{a,t‐1}}{\sum_{a^{\prime }=1}^{a^{\prime }=a\max}{\text{MAT}}_{a^{\prime },t‐1}N_{a^{\prime },t‐1}}$$

where Rec*_t_* is the recruitment at year *t*, MAT*_a_*_,_ *_t_*_−1_ the proportion of mature at age *a* and time *t* − 1 and *N_a_*_,_ *_t_*_−1_ the abundance for age *a* at time *t* − 1. Note that our model does not take into account the difference of egg productivity between age classes but only the maturity in percent.

We built annual transition matrices *A_t_* independently for two sets of data for NEA cod (1913–1999 and 1946–2016) and the two sets for NSS herring (1907–1998 and 1950–2015).

As a convenient way to summarize the year‐specific information contained in the transition matrices, we calculated their dominant eigenvalue (*λ*_1_). We interpret the dominant eigenvalue as a proxy for population growth for the given year. This interpretation is supported by a positive correlation between the eigenvalue and per capita growth rate (Figure S1). However, we note that the two measures of population growth do not necessary capture the same dynamics as the eigenvalue accounts for the age structure, while the per capita growth is a more aggregated measure. Specifically, we get four time series of *λ*_1_ *_t_* with the same scale and consequently directly comparable, which for convenience were log‐transformed *λ*_1_ (ln(*λ*_1_) fluctuating around 0). Since no data on recruitment at age 1 was available for NEA cod, we used as a recruitment proxy the age 3 data with a lag of 3 years and we set the survival between the first and second year and between the second and third year (*S*_2−1,_ *_t_* and *S*_3−2,_ *_t_*) to 1. It has previously been shown that the choice of this value does not substantially affect the estimate of *λ*_1_ (Rouyer et al., 2011). We tested this assumption by using for the two first years *S*_4−3,_ *_t_* = exp(−Mortality_4−3,_ *_t_*) reported by ICES (Table 3.17 p158 ICES, 2017). The use of this lower and variable survival while affecting the calculated value of *λ*_1_ did not affect the final model. For NSS herring, we used the age 0 recruitment provided by Toresen and Østvedt (2000) and the ICES outputs (Table 1).

### 2.3 Explanatory variables

As species potentially interacting with the cod, we used the immature herring biomass (*B*_herr_, annual difference between the total stock biomass and the spawning stock biomass (SSB),

Table 1. We used immature herring since adult herring are absent from the Barents Sea. For the herring models, we used the cod SSB (*B*_cod_) as potential competitor/predator (Table1).

The mean age in the spawning stock (MA), that was shown to affect population dynamic for both stocks (Durant & Hjermann, 2017; Rouyer et al., 2011), was calculated as the abundance‐weighted average of the ages of mature fish across age classes for both NEA cod and NSS herring:

$${\mathrm{MA}}_{\;t}=\frac{\sum_{a=a\min}^{a=a\max}a{\mathrm{MAT}}_{a,t}N_{a,t}}{\sum_{a=a\min}^{a=a\max}{\mathrm{MAT}}_{a,t}N_{a,t}}$$

with *a*min and *a*max being, respectively, the age of the youngest and the oldest age group contributing to the abundance of the spawners in year *t*, MAT*_a_*_,_ *_t_* the proportion of mature fish at age *a* and time *t*, and *N_e_*_,_ *_t_* the number of fish at age *a* and time *t*.

The fishing mortality (*F*) is given for age groups 5−10 (*F*_5–10_) and age groups 5−12 (*F*_5−12_) for cod and herring, respectively (Table 1). *F* represents the average removal of fish from the stock due to fishing activities and was shown to affect population dynamic for both stocks (Durant & Hjermann, 2017; Rouyer et al., 2011).

The sea temperature (ST) for the Barents Sea from 1921 until 2015 was obtained from the Kola meridian transect (33°30′E, 70°30′–72°30′N) collected by the Polar branch of the Russian Federal Institute of Fisheries and Oceanography (PB VNIRO formerly PINRO; (Tereschenko, 1996; http://www.pinro.ru/)). The Kola meridian transect intersecting the Murman Current in the south‐central Barents Sea, covers the inflow of Atlantic and Coastal water masses from the Norwegian Sea to the south‐eastern Barents Sea. This time series of temperature is representative of the Atlantic water masses in the south‐central Barents Sea (Ingvaldsen et al., 2003) and correlates positively with strong year‐classes of NEA cod (reviewed by Ottersen et al., 2014) and herring recruitment (Fiksen & Slotte, 2002). The annual values made available to us were calculated by averaging temperature horizontally along the transect (5 stations) and vertically from 0 to 200 m water depth (1 m, 10 m, 20 m, 30 m, 50 m, 75 m, 100 m, 150 m, and 200 m) (Bochkov, 1982; Tereschenko, 1996).

The station‐based winter index for the North Atlantic Oscillation (wNAO) calculated for the months of December, January, February, and March for the period 1921–2015 (http://www.cgd.ucar.edu/cas/jhurrell/indices.html, Hurrell & Deser, 2009).

### 2.4 Statistical analysis

Population structure, fishing, climate, and predation/competition effects on population change were investigated for both stocks for the two periods delimited by a population collapse (see collapse periods and Rouyer et al., 2011). In addition, we used environmental variables (i.e., wNAO and ST) that may influence several demographic variables simultaneously, including recruitment and age of maturity (Rouyer et al., 2011). The analysis was conducted using Generalized Additive Models (GAM), via the *mgcv* library in R 3.4.1 (R Core Team, 2014; Wood, 2006). GAM is a modeling technique which can be thought of as a generalization of ordinary multiple regression, where there may be both linear and nonlinear (smooth) effects of each explanatory variable (Wood, 2006). The GAM procedure automatically selects the degree of smoothing based on the Generalized Cross Validation (GCV) score. GCV is a proxy for the model's predictive performance. However, to avoid spurious and ecologically implausible relationships, we constrained the model to contain at maximum quadratic relationships, that is, we set the maximum degrees of freedom to 2 for each smooth term (i.e., *k* = 3 in the GAM formulation). We wanted a parsimonious model which described the response well but was as simple as possible. We used thin plate regression splines as smoothers.

All models started with a similar formulation:

$$\ln\left(\lambda_{1t}\right)=s_{1}\left({\text{MA}}_{t}\right)+s_{2}\left(F_{t}\right)+s_{3}\left({\text{ST}}_{t}\right)+s_{4}\left({\text{wNAO}}_{t}\right)+s_{5}\left(B_{t}\right)+\varepsilon_{t}$$

with *λ_t_* the dominant eigenvalue of the transition matrix *A_t_*, MA*_t_* the mean age of the spawning stock at time *t*, *F_t_* the fishing mortality at time *t*, ST*_t_* the Kola section sea temperature at time *t*, wNAO*_t_* the winter index for the North Atlantic Oscillation between *t* and *t* + 1, *B_t_* the biomass of the cod spawning stock (*B*_cod_ *_t_*) at time *t* for the herring models or of the juvenile herring (*B*_herr_ *_t_*) at time *t* for the cod models, and *ε_t_* an error term. The spline function is given as *s_i_* (*·*).

### 2.5 Model selection

We entered all candidate explanatory variables in the GAM model and conducted model selection using shrinkage. In the shrinkage approach to model selection, the smoother is modified so it allows insignificant variables to be shrunk to zero (i.e., effectively removed) as part of the smoothness selection (Wood, 2006). Thus, a reasonably optimal model is selected in a single step (i.e., all smoothers with 0 amount of smoothing are dropped simultaneously from the model). Before performing the model selection, we calculated the variance inflation factors (VIFs) between all explanatory variables to detect collinearity. Covariates with the highest VIFs were subsequently removed from the model until the highest VIF value was <3 (Zuur et al., 2007).

For one model (model for cod before the collapse), we found a temporal autocorrelation in the residuals of one‐year lag (using autocorrelation function *acf* in R). For the other three models, we did not detect any significant autocorrelation. To account for the autocorrelation in the residuals of the cod model before the collapse, we used an AR(1) model (autocorrelation term or first order, in our case ln(*λ*_1_ *_t_*_−1_)).

The quantification of an individual explanatory variable's contribution to a multiple regression model explaining the variation of the stock biomass was calculated using the package *relaimpo* (Grömping, 2006) using the “proportional marginal variance decomposition” (*pmvd*) (Feldman, 2005).

## 3 RESULTS

**FIGURE 2:**
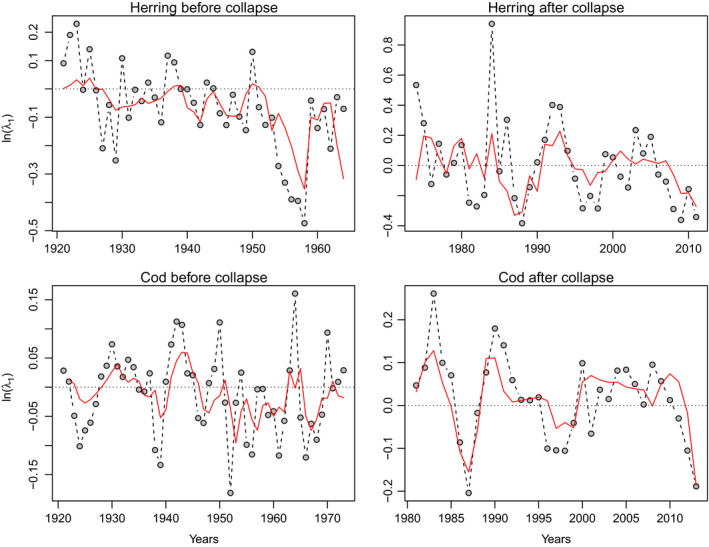
Model of the dominant eigenvalue *λ*_1_ for the periods before and after the collapse for Norwegian spring spawning (NSS) herring *Clupea harengus* and the Northeast Arctic (NEA) cod *Gadus morhua*. For each stock and each period is displayed the data used to obtain the model (points) and the corresponding GAM prediction (red line)

Time series of the dominant eigenvalue *λ*_1_ of the transition matrix for the NSS herring (*Clupea harengus*) and the NEA cod (*Gadus morhua*) are shown in the Figure 2 and Table S1.

**FIGURE 3:**
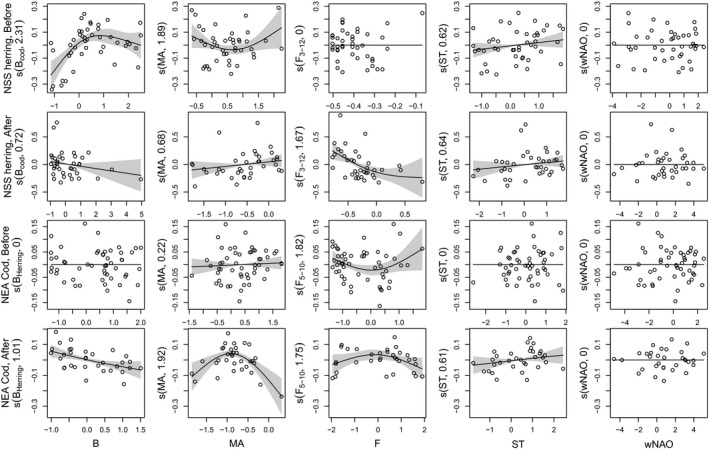
Model of the dominant eigenvalue *λ*_1_ of the annual transient matrices for the Norwegian spring spawning (NSS) herring *Clupea harengus* and the Northeast Arctic (NEA) cod *Gadus morhua* before and after a population collapse. The generalized additive models (GAM) are presented for each population. For each plot, the *x*‐axes show the covariate and the *y*‐axes the partial effect that each covariate has on the response variable. *s*(*X*, *y*) is the smoothing term, where *X* represents the explanatory variable and *y* is the estimated degrees of freedom (edf) of the smoothing term. Black line: smooth term effect of the considered covariate on the population growth with the pointwise 95% confidence interval around the mean prediction (shaded area). (s) Partial residuals calculated by adding the effect of the concerned covariate to the residuals; the model prediction at any given point is given by the sum of all partial effects plus a constant. *B*: cod spawning stock biomass (SSB) for the herring models and juvenile herring biomass for the cod models; *MA*: mean age of the spawning stock in years; *F*: fishing mortality; *wNAO*: winter North Atlantic Oscillation; *ST*: sea temperature at 0 to 200 m. *B*, *MA*, *F*, and *ST* were centered to 0 and normalized

The best stock‐specific GAM models of the dominant eigenvalue ln(*λ*_1_) are displayed on the Figure 2. The best GAM models of the dominant eigenvalue ln(*λ*_1_) for the NSS herring and the NEA cod before the collapse give us an insight of the change in interaction with other species and effects of environment and harvesting (Figure 3). To check the strength of dependency of ln(*λ*_1_) on fishing mortality (*F*) and the validity of our modeling approach we have run the four GAM models without including *s*_2_(*F_t_*). These four new models displayed similar effects (direction and strength) of the explanatory variables MA*_t_*, ST*_t_*, wNAO*_t_*, and B*_t_* than the full models (see Figure S2). This indicates that their effect on ln(*λ*_1_) was independent of the effect of *F*.

### 3.1 Norwegian spring spawning herring models

Before the collapse (Figure 3 first row), three explanatory variables are retained in the final model (Deviance (Dev) = 44.5%). The cod SSB (*B*_cod_) shows a positive effect (*p* < .001), mean age of the spawning stock (MA) a positive but not significant effect (*p* = .08), and Kola sea temperature (ST) a positive but not significant effect (*p* = .08) on the herring ln(*λ*_1_). The positive effect of *B*_cod_ may indicate a covariation.

After the collapse (Figure 3 second row), four explanatory variables are retained in the final model (Dev = 34.7%). *B*_cod_ shows a negative but not significant effect (*p* = .11), MA a positive but not significant (*p* = .11), fishing mortality (*F*) a negative effect (*p* < .01) and ST a positive but not significant (*p* = .14) on the herring ln(*λ*_1_).

### 3.2 Northeast Arctic cod models

Before the collapse (Figure 3 third row), two explanatory variables are retained in the final model (Dev = 26.5%). MA and *F* show a positive but not significant effect (*p* = .26 and *p* = .06, respectively) on the cod ln(*λ*_1_).

After the collapse (Figure 3 fourth row), four explanatory variables are retained in the final model (Dev = 61.5%). The juvenile herring biomass (*B*_herr_) shows a negative effect (*p* < .01), MA a positive then a negative effect after MA = 6.7 years (*p* < .01), F a not significant effect (*p* = .08), and ST a not significant positive effect (*p* = .13) on the cod ln(*λ*_1_).

**FIGURE 4:**
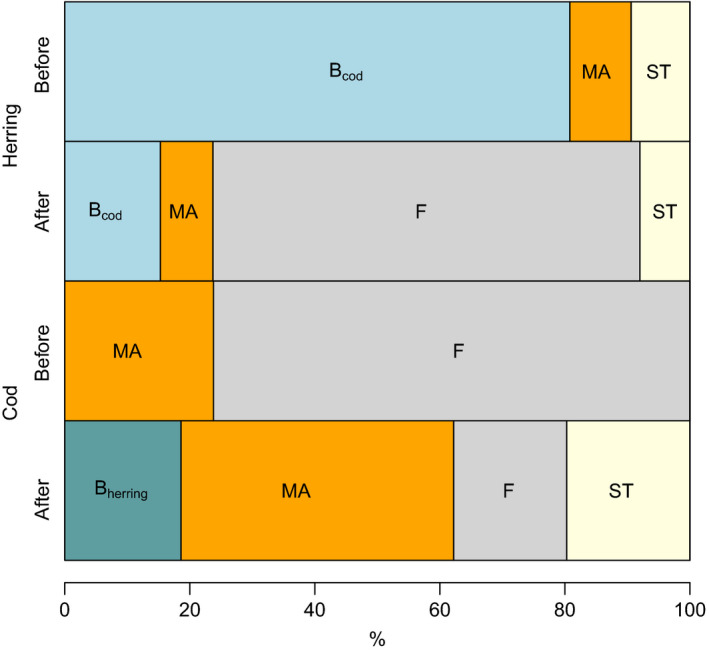
Relative importance of regressors (using proportional marginal variance decomposition, see methods) for each model in percent before and after a population collapse for herring and cod. *B*: cod SSB for the herring models or juvenile herring biomass for the cod models; *MA*: mean age of the spawning stock; *F*: fishing mortality; *ST*: sea temperature at 0 to 200 m; *wNAO*: winter North Atlantic Oscillation

The relative importance of regressors for each model in percentage is given in Figure 4. For the herring models, the variable explaining the most variation in ln(*λ*_1_) before the collapse is the cod SSB (*B*_cod_) replaced after the collapse by the fishing mortality (*F*). For the cod models, the variable explaining the most variation before the collapse is the fishing mortality (*F*) replaced after the collapse by the mean age of the spawning stock (MA).

## 4 DISCUSSION

Climate forcing (Drinkwater et al., 2010) and overexploitation of resources (Brander et al., 2010) have both been identified as the dominant factors affecting biodiversity and abundance of marine species. Since the beginning of the 20th century, several fish stocks and associated fisheries have collapsed (Branch et al., 2011; Hilborn, 2007). While environmental effects are generally stronger on younger stages, fishing primarily affects larger and older individuals. The combination of the two results in an increased variability in abundance and a greater risk of collapse (Sguotti et al., 2019). In addition, exploitation may alter population characteristics in such a way that the nature of response to environmental variability in a population will change (Planque et al., 2010; Rouyer et al., 2011). In other words, there are multiple feedback loops that might interact with each other potentially leading to stock collapses (Bakun & Weeks, 2006) with effects on the ecosystem that are still not completely understood.

By comparing the effect of different variables on ln(*λ*_1_) (a proxy of the changes occurring in the population between the two years), before and after a population collapse, in NSS herring (*Clupea harengus*) and NEA cod (*Gadus morhua*), we were able to explore the effect of such collapse on the population dynamics. There is no long time continuous and consistent time series survey data readily available that could be used to compare the dynamics before or after the collapse for these stocks (particularly before the collapses). Our study then relies on outputs from four virtual population analysis (VPA) models (Shepherd & Pope, 2002); a cohort modeling technique commonly used in fisheries science for stock assessment and reconstruction of historical fish numbers. VPAs are based on different sources of information (fisheries catch and scientific survey) and take into account the sampling intensity thus, while imperfect, give one of the best available estimate of historical population structure and numbers. However, each VPA has its particularities linked to data quality and specific methods. To reduce the potential impact of such issues, we calculated annual transition matrices independently for each VPA time series thus giving comparable time series of population dynamical properties before and after the collapse (see Table S1).

Previous studies have demonstrated that the realized population growth rate may be sensitive to change in the population structure linked, for example, to overfishing (Rouyer et al., 2011) associated with changing effects of sea temperature and fishing mortality on elasticity to recruitment (Durant & Hjermann, 2017). In this study, we extend these results by looking at the effect of a major decline in abundance on species interactions. Our results indicate that a population collapse in addition to an altered response to abiotic environmental variations (Rouyer et al., 2011) may lead to altered trophic interactions with subsequent potential effects at the ecosystem level. In other words, while the stocks have regained the abundance from before the collapse, the system is potentially quite different (Figure 4).

The explanatory power of our models is ranging from 26.5 to 61.5% (as expressed by the deviance) indicating that they are reasonably good for the type of analyze conducted (Figure 2). A reason for this somewhat low explanatory power could be the relatively high level of noise in the data, in particular for the herring before the collapse. For instance, a major part of the fishery before 1977 targeted immature herring (0–2 years old, Toresen & Østvedt, 2000) that may have altered the size composition of the stock. Another reason could be that we were unable to account for important explanatory variables. For example, it would have been useful to include the capelin *Mallotus villosus* abundance in our analysis, a key species in the Barents Sea (Hjermann et al., 2007). Unfortunately, capelin data are available only from 1974 and the comparison of its effect before/after the collapse is not possible. We can speculate that, following the absence of negative effect of herring abundance on cod before the cod collapse, the link herring‐capelin‐cod was potentially not as strong as it is currently (Hjermann et al., 2007). Since 1984, the NEA cod diet (fish sized 20–90 cm) is composed on average of only about 3% of herring and about 33% of capelin (Holt et al., 2019). Conversely, recent works on historical stomach's contents of NEA cod in the Barents Sea indicate that before the collapse, the herring was much more abundant than capelin in the cod diet (Townhill et al., 2015, 2021). Such diet difference is another indication of the change of the link herring‐capelin‐cod after the collapses. However, the absence of positive effect of herring on cod before the collapse may indicate that the juvenile herring was not a main prey for cod in the Barents Sea either despite a relatively high occurrence of herring in cod diet in the 1930s (see Figure S3 for herring abundance in the cod diet over the years) (Durant et al., 2014; Holt et al., 2019). This is confirmed by the positive effect of cod abundance on herring ln(*λ*_1_) before the collapse indicating a covariation. The absence of a negative effect of cod on herring before the collapse may also be explained by the very high abundance of herring relatively to the cod. However, note that the covariation was reduced at high cod abundance (Figure 3). Since the abundance of both herring and cod increased at the same time, explaining the observed covariation, the predation of cod on herring may not have been strong enough to be captured by the statistical analysis. On the reverse, after the collapse, the biomass of cod relatively to the abundance of herring was nearly quadrupled due to some years with very low herring abundance and recent years with very high cod biomass (Table S1).

Atlantic cod is considered a major predator of herring (Hamre, 1988, 1994; Link et al., 2009), particularly for the juvenile stage (de Barros & Toresen, 1998; Johansen et al., 2004), and we thus expected to observe a negative effect on herring population. However, we only observed, albeit not significantly, a negative effect of cod on herring after the herring collapse. In addition, a recent work shows a relatively little predation of cod on herring in the Barents Sea (Holt et al., 2019) which fit with an expected predation pressure relief after the cod stock collapses (e.g., for Gulf of Maine–Georges Bank Atlantic herring; Overholtz & Link, 2007). Furthermore, the change of cod diet toward a higher capelin diet after the 70s (Townhill et al., 2015, 2021) may also have led to a predation pressure relief.

On the other hand, the herring is also a major predator of capelin larvae in the Barents Sea (Hallfredsson & Pedersen, 2009; Hjermann et al., 2010). In periods of good herring recruitment, the recruitment of capelin population in the Barents Sea, a main prey item for cod (ICES, 2012; Johansen et al., 2004; Mehl, 1989), is strongly reduced due to herring predation on capelin larvae (Hamre, 1994). In the recent years, cod population growth was found to be positively related to the abundance of capelin (Durant et al., 2008). An increase of the herring predation on capelin would explain the negative effect of herring on the cod population (Figure 3) as would an increase of herring predation on cod eggs (Akimova et al., 2019; Segers et al., 2007). Alternatively, herring larvae being less sensitive to food depletion than cod larvae (Folkvord et al., 2009) they may outcompete cod larvae for food in years of high herring abundance (Hjermann et al., 2013) since their larvae are drifting along a similar route toward the Barents Sea (Vikebø et al., 2011). In the Georges Bank, predation by herring on cod early life stages was shown to delay the population rebuilding (Collie et al., 2013). Similar results have also been reported for interactions between cod and herring in the North Sea (Essington et al., 2014; Fauchald, 2010). However, this mechanism may not have prevented NEA cod rebuilding after its collapse (1974 onward) since the NSS herring stock was at this time still low in abundance as a result of the herring collapse in the late 1960s. During the same period, food in the Barents Sea, that is capelin stock, was also highly abundant (Durant & Hjermann, 2017). Conditions may have thus been favorable for a relaxed competition among surviving NEA cod, leading to an increase in food intake and hence increased somatic growth and reproduction (Van Leeuwen et al., 2008).

Many marine ecosystems are increasingly susceptible to sudden nonlinear transformations due to climate warming (Hoegh‐Guldberg & Bruno, 2010). Nonadditive effect of the environment (i.e., climate) on population dynamics has been observed in both terrestrial (Stenseth et al., 2004, 2015) and marine systems (Ciannelli et al., 2013; Dingsør et al., 2007) and may lead to different population equilibrium (Durant et al., 2020). This is particularly true for the Atlantic cod (Fauchald et al., 2011; Frank et al., 2011; Scheffer et al., 2001; Sguotti et al., 2019; Vasilakopoulos & Marshall, 2015). Using mass‐balance models, Bundy (2005) have shown that the fishing‐induced collapse of the cod stock led to a change in the structure of the eastern Scotian Shelf ecosystem (see also Bundy et al., 2009). However, the predator structure in Barents Sea system did not change as the cod remaining the main predator after the collapse, its stock having recovered.

In their study on cod and herring, Rouyer et al. (2011) considered the change in the realized population growth as a continuous variable and explored over time the effect of the population structure, fishing intensity, and sea temperature changes on its variation. In our study, we explored the change occurring to the population before and after a major decline in abundance considering the periods independently of each other (i.e., nonlinear) and looked at the difference in the resulting species interaction. We found that the collapse of the population may have led to more structural change in the ecosystem than expected, as the species interaction has changed. Similar to Rouyer et al. (2011), we found that the cod stock is more sensitive to climate variation in the recent period compared to before the population collapse (Figure 3). However, note that the period after the collapse corresponds mostly to a period of sea temperature increase with strong effect on the Barents Sea structure (Fossheim et al., 2015). Part of the observed differences (e.g., increased sensitivity to climate variation, Figure 3) could be explained by climate warming and by the change in feeding opportunities. Keeping in mind that our models have a different formulation, our results for herring differ from Rouyer et al. (2011) as we found that the fishing mortality is affecting the population dynamic after the collapse and not before.

Marine ecosystems are subjected to a range of exploitation intensities that can lead to stock declines and even collapses (Worm et al., 2009). While management actions have achieved measurable effects by reduction of the exploitation rates (Dragesund et al., 2008; Ulltang, 1987), these actions are not always resulting in a stock regeneration (Collie et al., 2013; Hutchings & Rangeley, 2011) or ecosystem regeneration, new ecological baseline may have developed (e.g., Blenckner et al., 2015). Indeed, changes of population structure linked to overexploitation have been shown to affect how a population responds to climate and exploitation forcing (Brosset et al., 2019; Durant & Hjermann, 2017; Hidalgo, Rouyer, et al., 2012; Rouyer et al., 2011). In this study, we show that in addition, the occurrence of a collapse is creating a nonlinearity in the species interactions that may eventually impact the functioning of the food chain similar to what was observed for the effect of climate warming (Ciannelli et al., 2013; Dingsør et al., 2007; Durant et al., 2020).

## CONFLICT OF INTEREST

The authors have no conflicts of interest to report.

## AUTHOR CONTRIBUTIONS

**Joël M. Durant:** Conceptualization (lead); data curation (lead); formal analysis (lead); funding acquisition (equal); investigation (lead); methodology (lead); project administration (equal); validation (lead); visualization (lead); writing–original draft (lead). **Leana Aarvold:** Formal analysis (supporting); investigation (supporting); methodology (supporting); validation (supporting); visualization (supporting); writing–original draft (supporting). **Øystein Langangen:** Conceptualization (supporting); formal analysis (supporting); funding acquisition (equal); investigation (supporting); methodology (supporting); project administration (equal); writing–original draft (supporting).

## Supporting information

Supplementary Material

## Data Availability

Source and accessibility of the data used in this study are given in Table 1 and Table S1. wNAO at https://climatedataguide.ucar.edu/climate‐data/hurrell‐north‐atlantic‐oscillation‐nao‐index‐station‐based. Fish abundance data at http://www.ices.dk/community/groups/Pages/AFWG.aspx and see Table 1. Kola sea temperature at http://www.pinro.ru/labs/hid/kolsec22.php.
