# Supplementary material for: Stock collapse and its effect on species interactions: Cod and herring in the Norwegian‐Barents Seas system as an example
Source: Ecol Evol. 2021 Dec 1;11(23):16993–7004. doi: 10.1002/ece3.8336 (PMC8668721; doi:10.1002/ece3.8336)
Supplement: Supplementary file 1 — Supplementary Material [file ECE3-11-16993-s001.pdf]

Supporting information for

Stock collapse and its effect on species interactions: cod and herring in the Norwegian-Barents Seas system as an example

Joël M. Durant, Leana Aarvold, Øystein Langangen

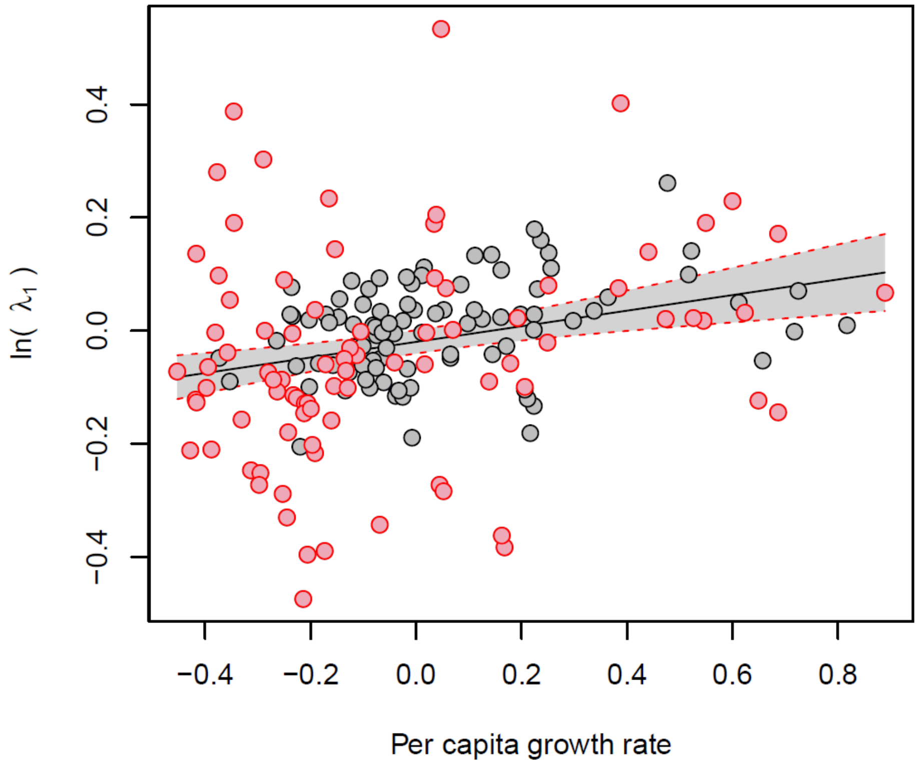

Figure 1. Relationship between the per capita annual growth rate ($r = (dN/dT)/N$) and the dominant eigenvalue of the annual transition matrices ($\ln(\lambda_1)$). In red are presented the data for the NSS herring and in grey for the NEA cod. The line is the significant linear relationship modelled ($p < 0.001$) with the standard errors interval. Note that the per capita growth was extremely variable for the herring. Some years, age 0 and age 1 abundances could be very high at the time of the sampling associated with a high mortality leading to an inflation of the total abundance retrained to one year only. The use of $\ln(\lambda_1)$ is likely a better representation of population growth.

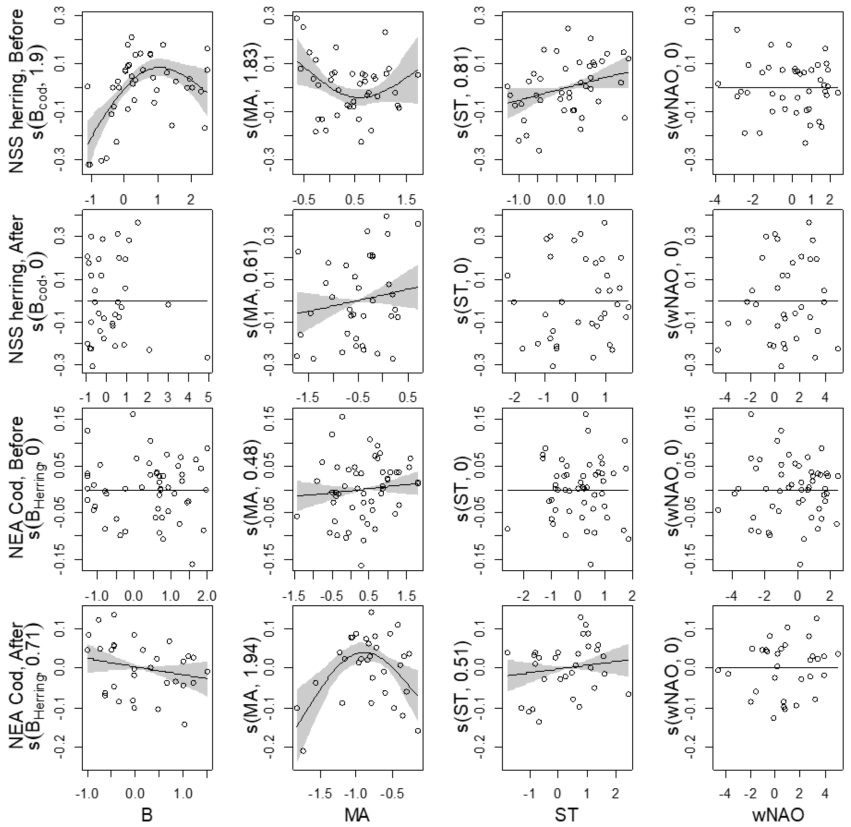

Figure 2. Model of the dominant eigenvalue λ_1 of the annual transient matrices for the Norwegian spring spawning (NSS) herring *Clupea harengus* and the Northeast Arctic (NEA) cod *Gadus morhua* before and after a population collapse. The generalized additive models (GAM) are presented for each population. For each plot, the *x*-axes show the covariate and the *y*-axes the partial effect that each covariate has on the response variable. s(X, y) is the smoothing term, where X represents the explanatory variable and y is the estimated degrees of freedom (edf) of the smoothing term. Black line: smooth term effect of the considered covariate on the population growth with the pointwise 95% confidence interval around the mean prediction (shaded area). (s) Partial residuals calculated by adding the effect of the concerned covariate to the residuals; the model prediction at any given point is given by the sum of all partial effects plus a constant. *B*: cod spawning stock biomass (SSB) for the herring models and juvenile herring biomass for the cod models; *MA*: mean age of the spawning stock in years; *wNAO*: winter North Atlantic Oscillation; *ST*: sea temperature at 0 to 200 m. *B*, *MA*, and *ST* were centred to 0 and normalized.

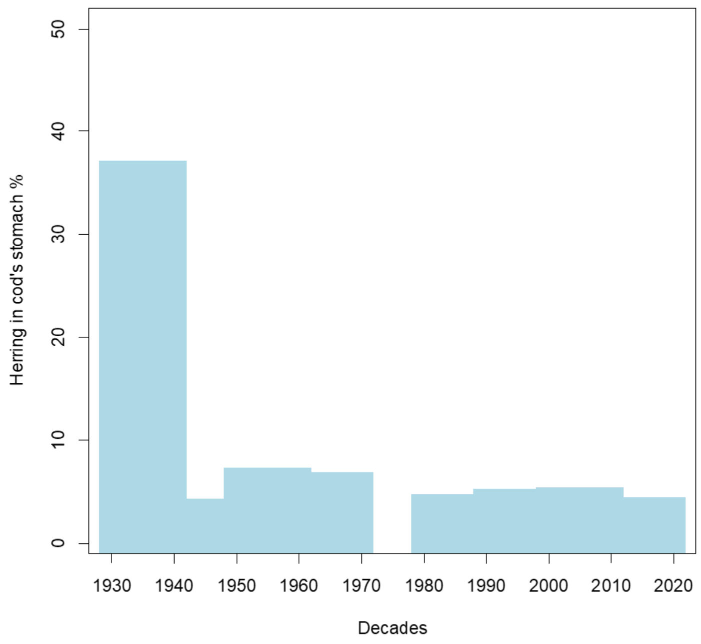

Figure 3. The percentage occurrence of herring in cod stomach each decade showing the highest presence of herring in the cod diet before than after the collapses of the 70s-80s. Data come from Townhill et al. 2020 (Townhill et al. 2020, Townhill et al. 2021).

Townhill BL, Holt RE, Bogstad B, Durant JM, Pinnegar JK, Johannesen E, Ottersen G (2020) Barents Sea Cod Diet Data. https://doi.org/10.21335/NMDC-213916938

Townhill BL, Holt RE, Bogstad B, Durant JM, Pinnegar JK, Dolgov AV, Yaragina NA, Johannesen E, Ottersen G (2021) Diets of the Barents Sea cod (Gadus morhua) from the 1930s to 2018. *Earth Syst. Sci. Data*, 13, 1361-1370.

Table 1a. Data used in the cod models before the collapse

Year	*Abundance*	*ln(λ₁)*	F_{5-10}	B_{herr}	*ST*	*wNAO*	*MA*
1921	1.76	0.03	-1.10	0.43	1.18	1.85	0.98
1922	2.16	0.01	-1.03	0.73	-0.01	1.73	0.85
1923	2.00	-0.05	-1.03	0.92	-0.32	-1.13	0.61
1924	1.87	-0.10	-0.97	1.23	-0.92	2.39	0.47
1925	1.70	-0.07	-1.23	1.44	0.24	0.11	0.37
1926	1.48	-0.06	-1.13	1.47	-1.09	1.72	0.57
1927	1.25	-0.03	-1.20	1.95	-0.48	0.63	0.86
1928	1.11	0.02	-1.16	1.32	-0.42	-1.03	1.06
1929	1.08	0.04	-1.03	0.79	-0.80	0.91	1.31
1930	1.13	0.07	-1.16	0.40	0.88	-0.16	1.63
1931	1.40	0.04	-1.13	0.21	0.32	-0.50	1.81
1932	1.87	0.02	-1.00	0.73	-0.73	0.25	1.81
1933	2.42	0.05	-0.93	0.62	0.74	0.86	1.56
1934	2.38	0.03	-0.90	1.30	0.95	0.97	1.01
1935	2.22	0.00	-0.97	1.09	0.89	-3.89	0.38
1936	2.13	-0.01	-0.77	0.91	0.20	0.72	0.18
1937	1.97	0.02	-0.70	0.61	1.30	1.79	0.30
1938	1.68	-0.11	-0.70	0.78	1.86	0.37	0.36
1939	1.62	-0.13	-0.70	1.89	1.67	-2.86	0.65
1940	1.98	0.01	-1.20	1.68	-0.59	-2.31	0.73
1941	1.82	0.07	-1.26	0.90	-1.29	-0.55	0.81
1942	1.66	0.11	-1.36	2.00	-1.23	1.48	0.75
1943	1.68	0.11	-1.36	1.28	0.86	0.61	0.68
1944	1.95	0.02	-1.36	0.69	0.60	1.64	0.83
1945	2.27	0.02	-1.29	0.69	0.10	0.27	1.00
1946	2.55	-0.05	-0.93	0.60	0.14	-2.71	1.20
1947	2.34	-0.06	-0.44	0.66	0.59	1.34	1.30
1948	2.11	0.01	-0.31	0.69	-0.53	1.87	1.25
1949	1.94	0.03	-0.24	0.44	0.64	1.40	0.85
1950	2.02	0.11	-0.24	0.44	1.74	-1.26	0.52
1951	2.53	-0.03	-0.11	0.77	1.09	0.83	0.35
1952	2.96	-0.18	0.31	1.58	0.45	0.18	0.30
1953	3.61	-0.03	-0.24	1.20	-0.42	0.13	0.14
1954	3.24	0.02	-0.15	1.82	1.78	-2.52	-0.03
1955	2.48	-0.10	0.35	0.69	0.60	-1.73	-0.36
1956	1.98	-0.12	0.68	-0.47	-1.00	1.52	-0.43
1957	1.90	0.00	0.25	-0.92	0.43	-1.02	-0.34
1958	1.78	0.00	0.28	-1.09	-0.86	-0.37	-0.15
1959	1.80	-0.05	0.41	-1.06	0.97	-1.54	0.04
1960	1.92	-0.04	0.15	-0.77	0.89	1.80	-0.39
1961	2.20	-0.12	0.64	-0.23	0.32	-2.38	-0.03

1962	2.14	-0.06	1.07	-0.25	0.22	-3.60	-0.38
1963	1.74	0.03	1.82	0.06	-1.29	-2.86	-0.74
1964	1.33	0.16	0.81	-0.04	0.30	-2.88	-0.20
1965	1.64	-0.05	0.38	-0.39	-0.46	-1.69	-0.12
1966	2.72	-0.12	0.31	-0.80	-2.62	1.28	-0.53
1967	3.30	-0.06	0.35	-0.80	-0.32	-1.04	-0.31
1968	2.55	-0.09	0.45	-1.09	-0.86	-4.89	-1.41
1969	1.65	-0.05	1.30	-1.28	-0.82	-1.89	-0.47
1970	1.03	0.09	1.04	-1.28	0.39	-0.96	-0.47
1971	0.96	0.00	0.54	-1.26	-1.00	0.34	-0.43
1972	1.65	0.01	0.84	-1.27	0.18	2.52	-0.87
1973	3.00	0.03	0.54	-1.28	0.89	1.23	0.16

Abundance = cod abundance in 10^9 individuals.
F = fishing mortality (mean = 0.4343023 and sd = 0.3045712)
B_{herr} = juvenile herring biomass in 10^6 tonnes (mean = 3.982698 sd = 3.082275)
ST = sea temperature at Kola in *°C* (mean = 3.972233 and sd = 0.4316446)
*wNA*O = winter North Atlantic Oscillation index
MA = mean age of the spawning stock biomass (mean= 8.659822 and sd= 1.373823)
F, B, ST and *MA* were centred to 0 ($x_{centered}$ = x – mean(x)) and normalized ($x_{normalized}$ = ($x_{centered}$/sd(($x_{centered}$)).

Table 1b. Data used in the cod model after the collapse.

Year	*Abundance*	$ln(\lambda_1)$	F_{5-10}	B_{herr}	*ST*	*wNAO*	*MA*
1981	0.61	0.05	0.65	-0.98	-1.77	0.80	-0.39
1982	0.55	0.09	0.91	-1.01	-0.79	3.42	-0.88
1983	0.48	0.26	0.72	-0.87	0.88	1.60	-0.98
1984	0.72	0.10	1.27	-0.44	0.00	-0.63	-1.09
1985	1.08	0.07	0.78	1.10	-0.79	0.50	-1.05
1986	1.87	-0.09	1.34	-0.46	-0.87	-0.75	-1.85
1987	1.69	-0.20	1.91	-0.05	-1.27	0.72	-1.78
1988	1.32	-0.02	1.59	-0.49	-0.68	5.08	-1.60
1989	0.97	0.08	0.09	-0.74	0.67	3.96	-1.26
1990	0.74	0.18	-1.31	-0.76	0.93	1.03	-0.83
1991	0.91	0.14	-1.56	-0.45	0.77	3.28	-0.47
1992	1.38	0.06	-0.91	-0.04	0.88	2.67	-0.32
1993	1.88	0.01	-0.27	0.45	-0.10	3.03	-0.44
1994	2.07	0.01	0.81	0.84	-0.52	3.96	-0.89
1995	1.82	0.02	0.73	1.20	0.45	-3.78	-0.98
1996	1.45	-0.10	0.65	1.03	-0.68	-0.17	-0.84
1997	1.44	-0.10	1.55	0.46	-1.05	0.72	-0.54
1998	1.73	-0.11	1.62	-0.34	-0.88	1.70	-0.42
1999	1.50	-0.04	1.62	-0.03	0.24	2.80	-0.35
2000	1.60	0.10	0.94	0.15	1.02	-1.90	-1.12

2001	1.62	-0.07	0.37	-0.02	0.74	0.76	-1.23
2002	1.49	0.04	0.11	0.32	0.61	0.20	-1.21
2003	1.66	0.02	-0.30	0.66	0.09	-0.07	-1.02
2004	1.38	0.08	0.41	0.99	1.34	0.12	-0.91
2005	1.50	0.08	0.40	1.21	1.38	-1.09	-0.79
2006	1.49	0.05	0.09	1.51	1.57	2.79	-0.77
2007	2.40	0.00	-0.93	1.00	1.63	2.10	-0.66
2008	2.94	0.10	-1.58	0.72	1.04	-0.41	-0.58
2009	2.88	0.06	-1.81	-0.04	1.13	-4.64	-0.85
2010	2.47	0.01	-1.78	-0.44	1.26	-1.57	-0.83
2011	2.34	-0.03	-1.83	-0.63	0.51	3.17	-0.65
2012	2.21	-0.11	-1.95	-0.64	2.42	-1.97	-0.20
2013	2.13	-0.19	-1.81	-0.64	1.48	3.10	0.31

Abundance = cod abundance in 10^9 individuals.
F = fishing mortality (mean = 0.63175 and sd = 0.2020467)
B_{herr} = juvenile herring biomass in 10^6 tonnes (mean = 2.558875 and sd =2.327398)
ST = sea temperature at Kola in *°C* (mean = 4.10125 and sd =0.5178666)
wNAO = winter North Atlantic Oscillation index
MA = mean age of the spawning stock biomass (mean= 7.839764 and sd= 1.226403)
F, *B*, *ST* and *MA* were centred to 0 ($x_{centered}$ = x – mean(x)) and normalized ($x_{nornalized}$ = ($x_{centered}$/sd(($x_{centered}$)).

Table 1c. Data used in the herring model before the collapse.

Year	*Abundance*	*ln(λ₁)*	*ST*	$B_{cod\ ssb}$	$F_{5\text{-}12}$	*wNAO*	*MA*
1921	62.65	0.09	1.17	0.01	-0.43	1.85	-0.27
1922	69.13	0.19	-0.02	0.10	-0.45	1.73	-0.53
1923	58.95	0.23	-0.33	0.21	-0.45	-1.13	-0.65
1924	85.66	0.00	-0.93	0.25	-0.45	2.39	-0.57
1925	124.33	0.14	0.23	0.48	-0.38	0.11	-0.41
1926	88.97	-0.01	-1.10	1.28	-0.36	1.72	-0.34
1927	124.62	-0.21	-0.48	1.44	-0.45	0.63	-0.28
1928	99.23	-0.06	-0.43	2.05	-0.50	-1.03	-0.22
1929	78.05	-0.25	-0.81	2.40	-0.48	0.91	-0.16
1930	79.52	0.11	0.86	2.48	-0.45	-0.16	0.11
1931	64.06	-0.10	0.30	1.89	-0.48	-0.50	0.39
1932	138.81	0.00	-0.74	0.78	-0.48	0.25	0.70
1933	90.08	-0.04	0.73	0.32	-0.48	0.86	0.94
1934	99.74	0.02	0.94	0.11	-0.50	0.97	0.87
1935	90.34	-0.03	0.88	0.23	-0.41	-3.89	0.76
1936	100.07	-0.12	0.19	0.98	-0.33	0.72	0.56
1937	89.74	0.12	1.29	1.16	-0.38	1.79	0.65
1938	77.31	0.09	1.84	0.79	-0.33	0.37	0.58

1939	137.59	0.00	1.65	0.84	-0.41	-2.86	0.35
1940	141.15	0.00	-0.60	0.34	-0.41	-2.31	0.43
1941	113.24	-0.05	-1.29	0.14	-0.45	-0.55	0.09
1942	111.00	-0.13	-1.24	-0.16	-0.48	1.48	0.01
1943	101.17	0.02	0.84	0.04	-0.50	0.61	0.04
1944	88.24	0.00	0.59	0.53	-0.50	1.64	0.16
1945	113.52	-0.09	0.09	1.56	-0.50	0.27	0.47
1946	110.04	-0.13	0.13	2.31	-0.48	-2.71	0.47
1947	92.89	-0.02	0.57	2.51	-0.50	1.34	0.68
1948	81.74	-0.10	-0.54	1.96	-0.45	1.87	0.75
1949	88.41	-0.15	0.63	0.89	-0.48	1.40	0.82
1950	76.40	0.13	1.73	0.46	-0.45	-1.26	1.16
1951	63.98	-0.06	1.07	0.29	-0.41	0.83	1.34
1952	165.24	-0.13	0.44	0.11	-0.38	0.18	1.36
1953	98.75	-0.10	-0.43	-0.35	-0.41	0.13	1.12
1954	81.12	-0.27	1.77	-0.23	-0.29	-2.52	0.62
1955	68.68	-0.33	0.59	-0.54	-0.33	-1.73	-0.09
1956	52.77	-0.39	-1.00	-0.71	-0.26	1.52	-0.22
1957	40.56	-0.40	0.42	-1.05	-0.31	-1.02	0.15
1958	31.99	-0.47	-0.87	-1.10	-0.31	-0.37	0.56
1959	24.71	-0.04	0.96	-0.22	-0.26	-1.54	0.89
1960	18.91	-0.14	0.88	-0.40	-0.24	1.80	1.28
1961	68.13	-0.07	0.30	-0.32	-0.38	-2.38	1.74
1962	49.73	-0.21	0.21	-0.67	-0.31	-3.60	2.25
1963	38.48	-0.03	-1.29	-1.05	-0.24	-2.86	2.15
1964	26.63	-0.07	0.29	-1.13	-0.07	-2.88	1.09

Abundance = Herring abundance in 10^9 individuals older than 2 years of age.
ST = sea temperature at Kola in *°C* (mean = 3.976494 and sd = 0.432821)
$B_{cod\ ssb}$ = cod spawning stock biomass in 10^5 tonnes (mean = 4.913314 and sd = 2.68918)
F = fishing mortality (mean = 0.2285714 and sd = 0.4147411)
wNAO = winter North Atlantic Oscillation index
MA = mean age of the spawning stock biomass (mean= 7. 809665 and sd= 1. 684318)
ST, *B*, *F* and *MA* were centred to 0 ($x_{centered}$ = x – mean(x)) and normalized ($x_{nornalized}$ = ($x_{centered}$/sd(($x_{centered}$)).

Table 1d. Data used in the herring model after the collapse.

Year	*Abundance*	$ln(\lambda_1)$	*ST*	$B_{cod\ ssb}$	$F_{5\text{-}12}$	*wNAO*	*MA*
1974	0.34	0.53	-0.14	-0.78	-0.20	1.63	-1.15
1975	2.35	0.28	0.65	-0.87	-0.62	1.37	-0.77
1976	2.43	-0.12	0.06	-0.75	-0.77	-2.14	-1.71
1977	1.89	0.14	-0.98	-0.19	-0.67	0.17	-1.76
1978	3.01	-0.06	-2.05	-0.55	-0.51	-2.25	-1.50
1979	2.63	0.02	-2.27	-0.76	-0.77	0.56	-1.19

1980	2.88	0.14	-0.87	-0.95	-0.74	2.05	-1.15
1981	4.03	-0.25	-1.76	-0.80	-0.43	0.80	-0.86
1982	2.74	-0.27	-0.75	-0.31	-0.55	3.42	-0.74
1983	2.38	-0.20	0.96	-0.40	-0.04	1.60	-0.63
1984	2.29	0.94	0.06	-0.57	-0.64	-0.63	-0.26
1985	60.55	-0.04	-0.75	-0.69	0.10	0.50	0.19
1986	26.69	0.30	-0.83	-0.77	1.67	-0.75	0.70
1987	27.32	-0.22	-1.25	-0.93	0.42	0.72	-1.78
1988	20.06	-0.38	-0.64	-0.72	0.80	5.08	-1.44
1989	17.51	-0.14	0.75	-0.54	-0.18	3.96	-1.06
1990	18.30	0.02	1.01	-0.25	0.40	1.03	-0.59
1991	25.35	0.17	0.85	0.90	-0.57	3.28	-0.22
1992	34.28	0.40	0.96	1.51	-0.55	2.67	0.04
1993	72.07	0.39	-0.04	1.10	-0.74	3.03	0.07
1994	99.08	0.10	-0.47	0.54	-0.44	3.96	0.19
1995	75.01	-0.09	0.52	0.33	-0.16	-3.78	0.16
1996	59.68	-0.28	-0.63	0.41	-0.36	-0.17	-0.44
1997	47.32	-0.20	-1.01	0.40	-0.22	0.72	-0.67
1998	43.49	-0.29	-0.84	-0.10	-0.32	1.70	-0.57
1999	34.35	0.08	0.30	-0.39	-0.14	2.80	-0.22
2000	57.76	0.05	1.11	-0.53	-0.17	-1.90	0.11
2001	58.04	-0.07	0.82	-0.16	-0.34	0.76	0.24
2002	44.45	-0.15	0.68	0.28	-0.26	0.20	0.30
2003	36.94	0.23	0.15	0.56	-0.37	-0.07	-0.33
2004	84.59	0.08	1.43	0.91	-0.40	0.12	-0.52
2005	70.59	0.19	1.48	0.63	-0.31	-1.09	-0.29
2006	93.09	-0.06	1.67	0.59	-0.28	2.79	-0.42
2007	67.59	-0.11	1.73	0.76	-0.37	2.10	-0.71
2008	62.52	-0.29	1.13	0.88	-0.21	-0.41	-0.57
2009	47.60	-0.36	1.21	2.12	0.04	-4.64	-0.41
2010	36.86	-0.16	1.35	3.04	-0.04	-1.57	-0.24
2011	37.23	-0.34	0.59	4.95	-0.07	3.17	-0.12

Abundance = Herring abundance in 10^9 individuals older than 2 years of age.
ST = sea temperature at Kola in °*C* (mean = 4.071116 and sd 0.5053002)
$B_{cod\ ssb}$ = cod spawning stock biomass in 10^5 tonnes (mean = 4.150035 and sd = 3.28577)
F = fishing mortality (mean = 0.3282641 and sd = 0.3964093)
wNAO = winter North Atlantic Oscillation index
MA = mean age of the spawning stock biomass (mean= 7.73914 and sd= 1.892556)
ST, *B*, *F* and *MA* were centred to 0 ($x_{centered}$ = x – mean(x)) and normalized ($x_{nornalized}$ = ($x_{centered}$/sd(($x_{centered}$)).

R codes to obtain the dominant eigenvalue time series

Example for the NSS herring before the collapse

```
# Abundance per age class
n.abundToresen = Table 6 in Toresen & Østveld Fish and Fisheries 2000. Stock
      numbers in millions individuals. Age-0 to age-15
      n.abundToresen[,2:17] <- n.abundToresen[,2:17]/1000 # transform in 10^9
      individuals

# Proportion of mature fishes
propmatToresen = Table 5 in Toresen & Østveld Fish and Fisheries 2000


#############################################################
# Calculate the transition matrices M for each year
#############################################################
# m1 = matrix of numbers
# R  = recruitment

m1 <- as.matrix(n.abundToresen[,3:16])# start from age-1 and remove age-15+
      category. Keep age-1 to age-14.
 ages <- c(1:14)                      # age classes
 mat  <- propmatToresen [,3:16]       # same as m1
 mat  <- as.matrix(mat)
 R    <- m1[,1]                       # number of age-1

nc   <- dim(m1)[2]                    # number of columns
nr   <- dim(m1)[1]                    # same as number of year
M.hT <- array(0, dim=c(nc,nc,nr))     # define a 3D matrix


for (t in 2:nr) {
  M <- matrix(0,nc,nc)    # create a 2D matrix M for transition t-1 to t
  maturity <- c(as.numeric(as.vector(as.character(mat[(t-1),]))))
  n.sp <- (m1[(t-1),]*maturity)       # number of spawners
  M[1,] <- as.double((R[t]*maturity)/sum(n.sp))

  for (i in 2:nc) {
    if (m1[(t-1),(i-1)]>0) {
        M[i,(i-1)] <- m1[t,(i)]/m1[(t-1),(i-1)]
    } else {
        M[i,(i-1)] <- 0
    }
  }
  M.hT[,,t-1] <- M         # put the 2D matrix M in a 3D matrix M.hT
}
rm("ages","M","n.sp","nr","nc","R","m1","mat") # clean the variables


#############################################################
## Work on the dominant eigenvalue
#############################################################
```

```
dim(M.hT)
# [1] 14 14 92
# 92 matrices 14*14
Lambda1 <- rep(NA,dim(M.hT)[3])
for (i in 1: dim(M.hT)[3]){
   S <- eigen(M.hT[,,i])  # select the 1st eigenvalue for each matrix in M.hT
   Lambda1[i] <- log(Re(S$values[1])) # log-transform λ1
   rm(S)
   };rm(i)

# Save the results with corresponding years.
# To Year=1981 corresponds ln(λ1) for the transition Matrix years 1981-1982
dataHerringbBefore <-
         as.matrix(cbind(n.abundToresen$Year, c(Lambda1[-dim(M.hT)[3]],NA)))
```
